# Molecular evolution and genetic variations of  V and W proteins derived by RNA editing in Avian Paramyxoviruses

**DOI:** 10.1038/s41598-020-66252-x

**Published:** 2020-06-12

**Authors:** Pachineella Lakshmana Rao, Ravi Kumar Gandham, Madhuri Subbiah

**Affiliations:** National Institute of Animal Biotechnology, Hyderabad, 500032 Telangana India

**Keywords:** Phylogenetics, Evolution, Molecular evolution, Virology, Viral evolution

## Abstract

The newly assigned subfamily *Avulavirinae* in the family *Paramyxoviridae* includes avian paramyxoviruses (APMVs) isolated from a wide variety of avian species across the globe. Till date, 21 species of APMVs are reported and their complete genome sequences are available in GenBank. The APMV genome comprises of a single stranded, negative sense, non-segmented RNA comprising six transcriptional units (except APMV-6 with seven units) each coding for a structural protein. Additionally, by co-transcriptional RNA editing of phosphoprotein (*P*) gene, two mRNAs coding for accessory viral proteins, V and W, are generated along with unedited P mRNA. However, in APMV-11, the unedited mRNA codes for V protein while +2 edited mRNA translates to P protein, similar to members of subfamily *Rubulavirinae* in the same family. Such RNA editing in paramyxoviruses enables maximizing the coding capacity of their smaller genome. The three proteins of *P* gene: P, V and W, share identical N terminal but varied C terminal sequences that contribute to their unique functions. Here, we analyzed the *P* gene editing site, V and W sequences of all 21 APMV species known so far (55 viruses) by using bioinformatics and report their genetic variations and molecular evolution. The variations observed in the sequence and hexamer phase positions of the *P* gene editing sites is likely to influence the levels and relative proportions of P, V and W proteins’ expressions which could explain the differences in the pathogenicity of APMVs. The V protein sequences of APMVs had conserved motifs similar to V proteins of other paramyxoviruses including the seven cysteine residues involved in MDA5 interference, STAT1 degradation and interferon antagonism. Conversely, W protein sequences of APMVs were distinct. High sequence homology was observed in both V and W proteins between strains of the same species than between species except in APMV-3 which was the most divergent APMV species. The estimates of synonymous and non-synonymous substitution rates suggested negative selection pressure on the V and W proteins within species indicating their low evolution rate. The molecular clock analysis revealed higher conservation of  V protein sequence compared to W protein indicating the important role played by V protein in viral replication, pathogenesis and immune evasion. However, we speculate the genetic diversity of W proteins could impact the degree of pathogenesis, variable interferon antagonistic activity and the wide host range exhibited by APMV species. Phylogenetically, V proteins of APMVs clustered into three groups similar to the recent classification of APMVs into three new genera while no such pattern could be deciphered in the analysis of W proteins except that strains of same species grouped together. This is the first comprehensive study describing in detail the genetic variations and the molecular evolution of *P* gene edited, accessory viral proteins of Avian paramyxoviruses.

## Introduction

Avian paramyxoviruses (APMVs) are a group of paramyxoviruses known to infect a variety of bird species across the globe. Till date, 21 species of APMVs have been identified and the list is expected to grow with increase in viral surveillance in wild and domesticated birds. With recent ICTV 2019 classification, these viruses now belong to three genera, *Metaavulavirus* (APMV-2, -5, -6, -7, -8, -10, -11, -14, -15 and -20), *Orthoavulavirus* (APMV-1, -9, -12, -13, -16, APV-A, APV-B and APV-C) and *Paraavulavirus* (APMV-3 and -4) within the new subfamily *Avulavirinae* under family *Paramyxoviridae* in the order *Mononegavirales*^1^. APMV-1 to -9 were isolated before 1980, APMV-10 to -13 were identified by 2015 and all the other APMVs were reported in the recent years^2^.

Avian Paramyxoviruses are enveloped with a single stranded, non-segmented, negative sense RNA genome of size 13 to 17 kb^2^. The prototype virus, APMV-1 of genus *Orthoavulavirus*, also well known as Newcastle disease virus or NDV, is the most extensively studied virus in this group. NDV causes severe economically important disease in poultry. There are five pathotypes of NDV based on the clinical signs exhibited by infected chickens: (a) viscerotropic velogenic or highly virulent, pantropic NDV causing severe mortality (b) neurotropic velogenic or highly virulent NDV, specifically causing neurological illnesses and high mortality (c) mesogenic or moderately virulent NDV, with mortalities as high as 50% and reducing egg production (d) lentogenic–either respiratory or enteric type NDV, low virulence and causing low reduction in egg production and (e) asymptomatic or avirulent NDV. However, the pathotype classification is not always clear-cut^3^. There are more than thousand strains of NDV which have been isolated, sequenced and found to exhibit wide spectrum of virulence. The viral RNA genome encodes for six genes arranged in tandem, each coding for six structural proteins, 3’-N-P-M-F-HN-L-5’^4,5^. N is nucleocapsid protein, each N protomer is known to bind exactly 6 nucleotides of genomic and antigenomic RNA of most paramyxoviruses thus imposing a hexamer phase on the entire RNA genome. In nature, the genomic length of paramyxoviruses is polyhexameric (6n + 0) which is found to be necessary for efficient replication and this is called the ‘rule of six’^6,7^. N together with P, phosphoprotein and L, large polymerase protein, forms viral RNA dependent RNA polymerase complex essential for viral genome transcription and replication; M, matrix protein, is seen within the envelope, aids in virus assembly and budding; two viral glycoproteins, F, fusion protein and HN, hemagglutinin-neuraminidase protein, are studded on the envelope and assist with fusion of virus with host membrane and receptor binding, respectively^4^. NDV F protein is known as the virulence determinant; the virulent strains have unique multiple basic amino acids, at least three arginine (R) or lysine (K) residues, at fusion protein cleavage site starting at amino acid position 113, and a phenylalanine residue at position 117^3^. APMV-6 is also known to express an additional small hydrophobic (SH) protein from *SH* gene located between *F* and *HN* genes^8^. Further, by co-transcriptional RNA editing of *P* gene, two mRNAs, V and W are expressed^4,9–12^. Also, in certain paramyxoviruses, by a process of alternative transcription initiation in *P* gene (+1 reading frame), accessory C proteins are generated^13,14^. Thus by these mechanisms, paramyxoviruses are able to efficiently utilize over 95% of their small RNA genome for expression of viral proteins^12^.

The *P* gene carries a slippery sequence, a stretch of adenosine (A) nucleotides and guanosine (G) nucleotides called the ‘editing site’ where insertions of 1 G or 2 G nucleotides occur during transcription of *P* gene by the stuttering viral polymerase that reiteratively reads the template base^12,15^. A single G nucleotide addition leads to +1 frameshift in the ORF, generating V mRNA with a frequency of 25 to 35% and two G nucleotides addition leads to +2 frameshift in the ORF generating W mRNA with a frequency of 2 to 8.5% and the unedited mRNA (60-70%) codes for P protein in NDV^12,16^. Among the paramyxoviruses, members of the subfamily *Rubulavirinae* and APMV-11 of genus *Metaavulavirus*, encode V protein from their unedited transcript, while P protein is coded by +2 frameshift and W protein is expressed by +1 frameshift^17,18^. The resulting three mRNAs from *P* gene (P, V and W mRNAs) share common N terminal sequences and differ both in length and amino acid composition in their C terminal region. Their specific functions are dictated by their unique C terminal sequences. Studies on V protein of APMV-1 and other paramyxoviruses have revealed that V protein is multifunctional, targets STAT1 degradation, interferes with MDA5, is interferon antagonist^19–22^, inhibits apoptosis^23,24^, assist in viral replication^20,25^ and plays important roles in tissue tropism, virulence determination^11,20,26^ and host range restriction^21^. On the other hand, there is very limited information about the function of W protein. The W protein of Nipah virus has been shown to impact viral pathogenesis and support the virus to evade the host immunity^27–29^. In APMV-1, the nuclear localization of W protein and its incorporation into the virion has been recently reported^16,30^.

The complete genome sequences of all 21 species of APMV have been described individually^17,31–62^. A comprehensive comparative analysis of complete genome and structural genes of 20 species of Avulaviruses has been recently published^2^. Three clades of viruses were concluded based on phylogenomic analysis of 20 APMVs: Clade I included APMV-2, -5, -6, -7, -8, -10, -11, -14, -15 and -20 (currently classified under new genus *Metaavulavirus*), clade II comprised of APMV -1, -9, -12, -13, -16, APV-A, -B and -C (currently assigned under genus *Orthoavulavirus*) and clade III included APMV-3 and -4 (currently under new genus *Paraavulavirus*)^1,2^. One of the viruses, previously classified as APMV-17 (South Korean “avian paramyxovirus 17”) has now been proposed as a separate species (APMV-21) based on phylogenies of complete genomes, complete *F* and *L* genes, PASC and STD analysis^2,63^. Nevertheless, very little is known about the *P* gene edited accessory viral proteins of APMVs. We have examined and analyzed 55 viruses belonging to all 21 APMV species identified till date and discuss here the genetic diversity and molecular evolution of *P* gene edited proteins, V and W.

## Materials and methods

### Sequence information

The full length sequences of *P* gene available for all the 21 species of APMVs were obtained from National Center for Biotechnology Information (NCBI) (http://www.ncbi.nlm.nih.gov/). Additionally, publications reporting the complete genome sequence of these viruses were also referred for identification of *P* gene editing site, for prediction of sequences of  V and W proteins. A total of 55 viruses belonging to 21 species of APMVs were analyzed in this study which included (their GenBank accession numbers are provided in Table 1) four strains each of avirulent APMV-1, moderately virulent APMV-1 and highly virulent APMV-1; eight isolates of APMV-6; five isolates each of APMV-2 and APMV-8; four isolates of APMV-10; three isolates of APMV-13; two isolates each of APMV-3, APMV-4, APMV-5; one isolate each of APMV-7, APMV-9, APMV-11, APMV-12, APMV-14, APMV-15, APMV-16, APMV-20, APMV-21 and APV-A, APV-B and APV-C. Detailed information of these isolates along with metadata such as hosts, year and location of isolation in addition to their sequence information are provided in Table 1. The complete sequences of *P* gene ORF, V proteins and W proteins used in this study that were either directly collected from NCBI or derived by prediction using DNASTAR software suite are provided in Supplementary Files S1, S2 and S3.

**Table 1:** Detailed information on V and W proteins of APMV species and strains analyzed in this study.

**S.No**	APMV species		Genbank Id	Year of Isolation	Place of isolation	Isolated from	P ORF (Nt) /P Protein length (aa)	*P* gene ‘RNA editing site’ sequence (numbers indicate the position of nucleotide in the genome, editing site is bolded and hexamer phase of the start of the short C run in edit site is underlined)	Subunit hexamer phasing at the *P* gene mRNA editing site	V ORF (Nt) /V Protein length (aa)	W ORF (Nt) /W Protein length (aa)	No. of unique amino acids in the C terminal region of W protein
1	APMV -1 (NDV)	Velo-genic	KJ808820.1	2012	China	Pigeon	1188 /395	^2283^CGA**U UUUUCC C**CGG	5	720/239	684/227	94
2			FJ386394.2	2009	China	Chicken	1188 /395	^2277^CGA**U UUUUCC C**GGG	5	720/239	666/221	88
3			GU187941.1	1987	India	Chicken	1188/395	^2277^CGA**U UUUUCC C**GGG	5	720/239	531/196	62
4			JQ015296.1	2011	China	Chicken	1188/395	^2283^CGA**U UUUUCC C**GGG	5	720/239	534/177	43
5		Meso-genic	KX761866.1	2010	China	Duck	1188/395	^2277^CGA**U UUUUCC C**GGG	5	720/239	684/227	94
6			KT445901.1	1945	Palestine	Duck	1188/395	^2277^CGA**U UUUUCC C**GGG	5	720/239	666/221	88
7			AY562986.1	1993	USA	Anhinga	1188/395	^2283^UGA**U UUUUCC C**GGG	5	720/239	540/179	45
8			KJ736742.1	1998	Germany	Pigeon	1188/395	^2283^CGA**U UUUUCC CC**GG	5	720/239	414/137	3
9		Lento-genic	KM885162.1	2013	China	Duck	1200/399	^2277^CGA**U UUUUCC C**GGG	5	738/245	552/183	49
10			JN688862.1	2009	China	Chicken	1200/399	^2277^CGA**U UUUUCC C**GGG	5	738/245	552/183	49
11			JX193078.1	2009	China	Duck	1188/395	^2277^CGA**U UUUUCC C**GGG	5	720/239	540/179	45
12			AB524405.1	1991	Alaska	Goose	1200/399	^2277^CGA**U UUUUCC C**GGG	5	738/245	552/183	49
13	APMV - 2		HM159993.1	2006	England	Chicken	1200/399	^2089^CAA**UUU UUCCCC** UU	3	699/232	624/207	67
14			HQ896023.1	1999	China	Gallus gallus	1200/399	^2089^CAA**UUU UUCCCC** UU	3	699/232	624/207	67
15			EU338414.1	1956	USA	Chicken	1200/399	^2089^CAA**UUU UUCCCC** UU	3	699/232	624/207	67
16			HM159994.1	1980	Kenya	Gadwell	1200/399	^2089^CAA**UUU UUCCCC** UU	3	699/232	624/207	67
17			HQ896024.1	2001	China	Gallus gallus	1200/399	^2089^CAA**UUU UUCCCC** UU	3	699/232	624/207	67
18	APMV - 3		EU403085.1	1975	Netherland	PKT	1161/386	^2078^AAAAA **UUUCCC CC**G	4	747/248	378/125	14
19			EU782025.1	1968	USA	Turkey	1173/390	^2083^AAAAA**U UUCCCC C**G	3	759/252	384/127	12
20	APMV - 4		FJ177514.1	1975	China	Duck	1182/393	^2052^G AAAA**UU UCCCCC C**	2	675/224	414/137	2
21			JX133079.1	2010	South Africa	Alopochen aegyptiacus	1182/393	^2052^G AAAA**UU UCCCCC C**	2	675/224	414/137	2
22	APMV - 5		GU206351.1	1974	Japan	Budgerigar	1341/446	^2359^AA**UUUU UUCCC**G GG	3	834/277	564/187	5
23			LC168750.1	1975	Japan	Melopsitt-acus undulatus	1341/446	^2357^GU AA**UUUU UUCC**GG G	3	834/277	564/187	5
24	APMV - 6		JX522537.1	2011	China	Anas poecilorhyn-cha	1293/430	^2146^AG**U UUUUUC CCC**UU	6	825/274	489/162	4
25			KP762799.1	2013	Kazakhstan	Red-crested pochard	1293/430	^2146^AG**U UUUUUC CCC**UU	6	825/274	489/162	5
26			EU622637.2	1977	Hong Kong	Duck	1293/430	^2146^AG**U UUUUUC CCC**UU	6	807/268	534/177	5
27			AY029299.1	1998	Taiwan	Duck	1293/430	^2146^AG**U UUUUUC CCC**UU	6	807/268	534/177	20
28			KF267717.1	2011	China	Anas poecilorhyncha	1293/430	^2146^AG**U UUUUUC CCC**UU	6	825/274	489/162	20
29			KT962980.1	2009	Russia	Anas crecca	1293/430	^2139^AG**UU UUUUCC CC**UU	5	825/274	594/197	5
30			EF569970.1	2003	Russia	Domestic goose	1293/430	^2146^AG**U UUUUUC CCC**UU	6	807/268	534/177	40
31			GQ406232.1	2007	Italy	Duck	1293/430	^2134^AG**U UUUUUC CC**GCU	6	816/271	474/157	4
32	APMV - 7		FJ231524.1	1975	USA	Dove	1185/394	^2150^AA**UUU UUUCCC** AAU	4	753/250	378/125	1
33	APMV - 8		FJ215863.2	1976	USA	Canada goose	1218/405	^2095^UAA**UUU UUCCC**G GG	3	717/238	612/203	65
34			MF448515.1	2013	Kazakhstan	Little stint	1218/405	^2095^UAA**UUU UUCCC**G GG	3	717/238	519/172	34
35			JX901129.1	1978	Japan	Pintail duck	1218/405	^2095^UAA**UUU UUCCC**G GG	3	717/238	519/172	34
36			FJ619036.1	1976	USA	Canada goose	1218/405	^2095^UAA**UUU UUCCC**G GG	3	717/238	519/172	34
37			MF448514.1	2013	Kazakhstan	Whooper swan	1218/405	^2095^UAA**UUU UUCCC**G GG	3	717/238	519/172	34
38	APMV - 9		EU910942.1	1978	USA	Domestic duck	1260/419	^2319^GA**UU UUUUCC C**GAG	5	792/263	585/194	56
39	APMV - 10		HM755886.2	2007	United Kingdom	Penguin	1218/405	^2094^A GAA**UUU UUCCC**G UG	3	741/246	519/172	34
40			HM147142.3	2007	Falkland Islands	Penguin	1218/405	^2094^A GAA**UUU UUCCC**G UG	3	741/246	519/172	34
41			HM755887.2	2007	United Kingdom	penguin	1218/405	^2094^A GAA**UUU UUCCC**G UG	3	741/246	519/172	34
42			HM755888.2	2007	United Kingdom	penguin	1218/405	^2094^A GAA**UUU UUCCC**G UG	3	741/246	519/172	34
43	APMV - 11		JQ886184.1	2010	France	Common snipe	1344 /447(2 G insertion)	^2333^AA AA**UUCU UCCCCC** A	2	834/277 (V mRNA is the unedited transcript)	546/181(1 G insertion)	15
44	APMV - 12		KC333050.1	2005	Italy	Wigeon	1218/405	^2324^UGA**UU UUUUCC C**UAG	5	762/253	564/187	31
45	APMV - 13		KU646513.1	2013	Kazakhstan	Wild goose	1194/397	^2318^UGA**UU UUUUCC C**GUC	5	726/241	453/150	2
46			KX119151.2	2011	Ukraine	White-fronted goose	1194/397	^2318^UGA**UU UUUUCC C**GUC	5	726/241	453/150	2
47			LC041132.1	2000	Japan	Ansersp	1194/397	^2318^UGA**UU UUUUCC C**GUC	5	726/241	453/150	2
48	APMV - 14		KX258200.1	2011	Japan	Duck	1227/408	^2094^A AAA**UUU UCCCC**G GC	2	759/252	471/156	12
49	APMV – 15		KX932454.2	2012	Brazil	Calidrisfuscicollis	1248/415	^2039^AA **UUUUUC CC**CCCU G	6	759/252	438/145	2
50	APMV - 16		KY511044.1	2014	South Korea	Wild duck	1200/399	^2289^UGA**U UUUUUC CC**AGU	6	738/245	423/140	5
51	APV-A (APMV – 17)		KY452442.1	2014	Antarctica	Pygoscelispapua	1209/402	^2105^AA**UUUUUUCCC**CAGU	Hexamer phasing position not determined as their genome lengths did not conform to ‘rule of six’	771/256	492/163	12
52	APV-B (APMV – 18)		KY452443.1	2014	Antarctica	Pygoscelispapua	1179/392	^2116^AA**UUUUUUCCC**CAGU		711/236	531/176	35
53	APV-C (APMV – 19)		KY452444.1	2014	Antarctica	Pygoscelispapua	1134/377	^2153^GAA**UUUUUUCCC**AGU		666/221	393/130	4
54	APMV - 20		MF033136.1	2014	Kazakhstan	Gull	1296/431	^2171^AA AAUUUU CCCCCU C	1	792/263	498/165	17
55	APMV - 21		MF594598.1	2015	South Korea	Wild birds	1320/439	^2270^CGA**UU UUUUCC C**GUC	5	915/304	432/143	3

A summary on the viruses analyzed in this study with specifications of their P, V and W protein sequences.

Nt: nucleotides, aa: amino acids.

### Sequence alignment, comparison and prediction of conserved motifs/domains

Multiple sequence alignments of V and W proteins were performed using the TCOFFEE multiple alignment algorithm, mode ‘expresso’ and the sequence similarities were colored through ESPript^64–66^. All residues/amino acid positions mentioned in the results and discussion correspond to APMV-1 strain KJ808820.1, the strain that appears first in the alignment file. Individual sequences were also analyzed in NCBI’s interface, conserved domain (CD)-search^67^ and aligned sequences were run in DREME version 5.0.5 software^68^ to identify conserved motifs/domains. The intraclade amino acid percentage identity was estimated using Megalign software from DNASTAR.

### Prediction of Nuclear Localization Signal (NLS) and Nuclear Export Signal (NES) in V and W proteins

The nuclear localization signal (NLS) in V and W proteins of APMV species were identified using online tool, cNLS mapper with a cut-off score of 5.0 that predicted NLS specific to the importin αβ pathway^69^. The presence of nuclear export signal (NES) in V and W proteins of APMV species was predicted using online tool, NetNES 1.1 server that predicted leucine-rich NES using a combination of neural networks and hidden Markov models^70^ and using LocNES that predicted the classical NESs in CRM1 cargoes^71^.

### Phylogenetic analysis and evolutionary divergence

Phylogenetic analysis was performed using MEGA7 software. For drawing the phylogenetic trees, evolutionary history was inferred by using the Maximum Likelihood method with JTT matrix-based model^72^ for V proteins and Dayhoff matrix based model for W proteins^73^. For drawing the phylogenetic tree of V proteins, bootstrap consensus tree inferred from 500 replicates was taken to represent the evolutionary history of the taxa analyzed^74^. Branches corresponding to partitions, reproduced in less than 80% bootstrap replicates, were collapsed. The percentage of replicate trees in which the associated taxa clustered together in the bootstrap test (500 replicates) are shown next to the branches. Initial trees for heuristic search were obtained automatically by applying Neighbor-Joining and BioNJ algorithms to a matrix of pairwise distances estimated using a JTT model, and then topology with superior log likelihood value was selected. A discrete gamma distribution was used for V proteins tree to model evolutionary rate differences among sites (16 categories (+G, parameter = 2.4693)). The rate variation model allowed for some sites to be evolutionarily invariable ([+I], 5.67% sites). For drawing the phylogenetic tree of W proteins, a discrete gamma distribution was used to model evolutionary rate differences among sites (5 categories (+G, parameter = 1.1488)). The rate variation model allowed for some sites to be evolutionarily invariable ([+I], 0.70% sites). Analysis of both the trees involved 55 amino acid sequences. All positions containing gaps and missing data were eliminated. There were a total of 141 and 71 positions in the final dataset for drawing V and W proteins’ phylogenetic trees, respectively.

The estimates of evolutionary divergence over sequence pairs between groups were analyzed for V and W proteins of all 21 APMV species using MEGA7^75^. Briefly, based on the maximum likelihood fits of 56 different amino acid substitution models, the final analyses were conducted in MEGA7 using JTT matrix-based model^72^ for V proteins and Dayhoff matrix based model^73^ for W proteins. The rate variation among sites was modeled with a gamma distribution (shape parameter = 1). The analysis included 55 amino acid sequences. All positions containing gaps and missing data were eliminated.

### Selection pressure analysis

The number of nonsynonymous substitutions per nonsynonymous site (dN), the number of synonymous substitutions per synonymous site (dS), and the dN/dS ratios for the nucleotide sequences of V and W proteins of all 21 species were analyzed for the entire sequence and also their shared N terminal and unique C terminal regions. The shared portion in the N-terminus of all the three proteins was considered up to the RNA editing site (KKG motif). The C terminal regions of  V and W proteins of all 21 species were considered after RNA editing site (KKG motif). The dN/dS ratio of 21 species of APMV nucleotide sequences were estimated by DnaSP v6.12.03 software^76^. The protein was considered under positive selection or diversifying when the dN/dS ratio is >1 and negative or purifying selection when dN/dS ratio <1.

### Evolutionary rate analysis

To estimate evolutionary rates of different APMV species in V and W nucleotide sequences, the substitution rate analysis was performed by BEAST v 1.10.4 software^77^. The substitution model GTR and site heterogeneity model G + I was found to be the best by MEGA7 and was used here to study the substitution rate of  V and W sequences. The tree prior coalescent, constant size was used for individual and all the species. The uncorrelated relaxed clock with lognormal was implemented. The MCMC chain 4 × 10^8^ cycles was used to reach the ESS value more than 200 to converge the data except for W proteins of APMV-8 strains where MCMC chain length of 2 × 10^8^ cycles was used. The final data analysis was performed using tracer v 1.7.1 software.

## Results

### RNA editing site and prediction of  V and W protein sequences

Previous reports suggested identical *P* gene RNA editing sites for APMV-1, -2, -4, -5, -6, -7, -8, -9, -10, -12, -13, -15, -20, APV -A, -B and –C and varied *P* gene editing site sequences for APMV-3, -11, -14 and -16. We observed the following conserved pattern in the RNA editing sequences among APMVs: U_3_C_6_ for APMV-4; U_4_C_4_ for APMV-14; U_4_C_5_ for APMV-20; U_5_C_3_ for APMV -1 (all strains except KJ736742.1 and KJ808820.1), APMV-8 and APMV-10; U_5_C_4_ for APMV -1 (strains KJ736742.1 and KJ808820.1) and APMV-2; U_5_C_6_ for APMV-15; U_6_C_3_ for APMV -5 (strain GU206351.1), APMV -7, -9, -12, -13, -16, -19, -21; U_6_C_2_ for APMV-5 (strain LC168750.1); U_6_C_4_ for APMV -6, -17, -18 and UUCUUC_5_ for APMV-11. Further, variations were observed in the cis-acting sequence at the editing site: the sequences immediately upstream of the editing site were^3^’AA in APMV -2, -3, -4, -5, -7, -8, -10, -11, -14, -15, -17, -18, -19, -20 and^3^’GA in APMV -1, -9, -12, -13, -16 and -21 (all orthoavulaviruses) while^3^’AG was conserved among APMV-6 strains.

The hexamer phase of the start of the template C run in the *P* gene editing site (Table 1) revealed that this position was conserved within each species except in APMV-3 and for one strain of APMV-6 (KT962980.1). The hexamer phasing positions for APV -A, -B, -C were not determined as their genome lengths did not conform to ‘rule of six’. The start of the C run was at hexamer position 1 for APMV-20; at hexamer position 2 for APMV -4, -11 and -14; at hexamer position 3 for APMV -2, -3 (EU782025), -5, -8 and -10; at hexamer position 4 for APMV-3 (EU403085) and APMV-7; at hexamer position 5 for APMV-1, one strain of APMV-6 (KT962980.1), APMV -9, -12, -13, and -21 and at hexamer position 6 for APMV -15, -16 and all strains of APMV-6 except one strain (KT962980.1).

All APMVs except APMV-11 expressed P protein from unedited mRNA. The editing site of APMV-11 resembled that of other paramyxoviruses that insert 2G for generating P mRNA or the ‘genomic V’ viruses^12^. The P protein of APMV-11 is derived from 2G nucleotides insertion, W protein from single G nucleotide addition and unedited mRNA expresses the V protein^17^. The V and W protein sequences of the other 20 APMV species were predicted by insertion of single G and two G nucleotides at the *P* gene RNA editing site, respectively.

### Amino acid sequence analysis: percentage identity and conservations

The V and W protein sequences of all 21 APMV species shared common N terminal region with P protein. The variations in the amino acid sequences were minimum within the first 60 amino acids in N terminal region. The N-terminal portions of P, V and W proteins of metaavulaviruses and orthoavulaviruses showed closer identity than paraavulaviruses. In ortho and paraavulaviruses, the N- and C- terminal regions showing high homology up to 93%. Metaavulaviruses showed 100% identity in all their C- and N- terminal regions. The C terminal region of W-protein sequences showed 0.0–100% identity, as both the amino acid composition and length variations at C-terminal portion for all the species were higher (Table 2). As described previously, the soyuz1 and soyuz2 motifs were observed within N terminal region in all APMVs except in APMV-3 strains^78^. Additionally, conserved domains (CD) were predicted in APMV-1 mesogenic strain Komarov (CD between aa 25 to 167) for large tegument protein UL36 (superfamily member PHA03247), in APMV-14 (CD between aa 31 and 144) for gene regulated by oestrogen in breast cancer- GREB1 (superfamily member, pfam15782) and in APMV-21 (CD between aa 53 and 120) for Tumor necrosis factor receptor superfamily member cd13415.

**Table 2:** Intraclade percentage amino acid sequence identity for P-gene products in APMV species.

	Genus *Orthoavulavirus*	Genus *Metaavulavirus*	Genus *Paraavulavirus*
P complete	31.8–99.5	22.6–100	24.6–91.9
V complete	13.5–99.2	26.1–100	18.6–92.9
V- N terminal	31.0–98.0	22.5–100	19.5–93.3
V- C terminal	30.3–98.9	26.3–100	27.0–92.1
W complete	26.3–99.5	19.6–100	14.8–92.7
W- N terminal	31.0–99.2	22.4–100	16.7–93.3
W- C terminal	0.0–100	0.0–100	50.0–100

P complete, V complete and W complete refer to the entire protein sequences. N terminal refers to N terminal region of respective proteins shared with P protein and C terminal refers to the unique C terminal region of respective proteins. The amino acid sequences of a particular protein of all virus strains, within the particular genus, were analyzed and the percentage amino acid identity was calculated using Lasergene DNASTAR MegAlign software.

### Comparison of V protein sequences of APMV species

The V protein of APV-C was the shortest (221 aa, MW: 23.73 kDa) and that of APMV-21 was the longest (304 aa, MW: 31.98 kDa) among the 21 APMV species. The length of V protein (in aa) conserved within species was as follows: APMV-2 strains (232 aa), APMV-4 strains (224 aa), APMV-5 strains (277 aa), APMV-8 strains (238 aa), APMV-10 strains (246 aa) and APMV-13 strains (241 aa). However, in APMV-1, -3 and -6, variation in the length of V protein was observed between strains within the same species. Between species, the similarity in the V protein length was observed as follows: V protein length of 252 aa was observed in APMV-14, -15 and one strain of APMV-3; APMV-11 and -5 showed 277 aa long V protein; APMV -9 and -20 had 263 aa long V protein while the V protein length of APMV-16 and two lentogenic strains of APMV-1 were 245 aa. The lowest amino acid identity (10.4%) was observed between V proteins of APMV-3 strain Wisconsin and APV-A. The lowest amino acid divergence was noticed between APV-B and APV-C (70.4) and both showed identity of 53.7% at amino acid level which was the highest between APMV species (Supplementary File S4).

**Figure 1:**
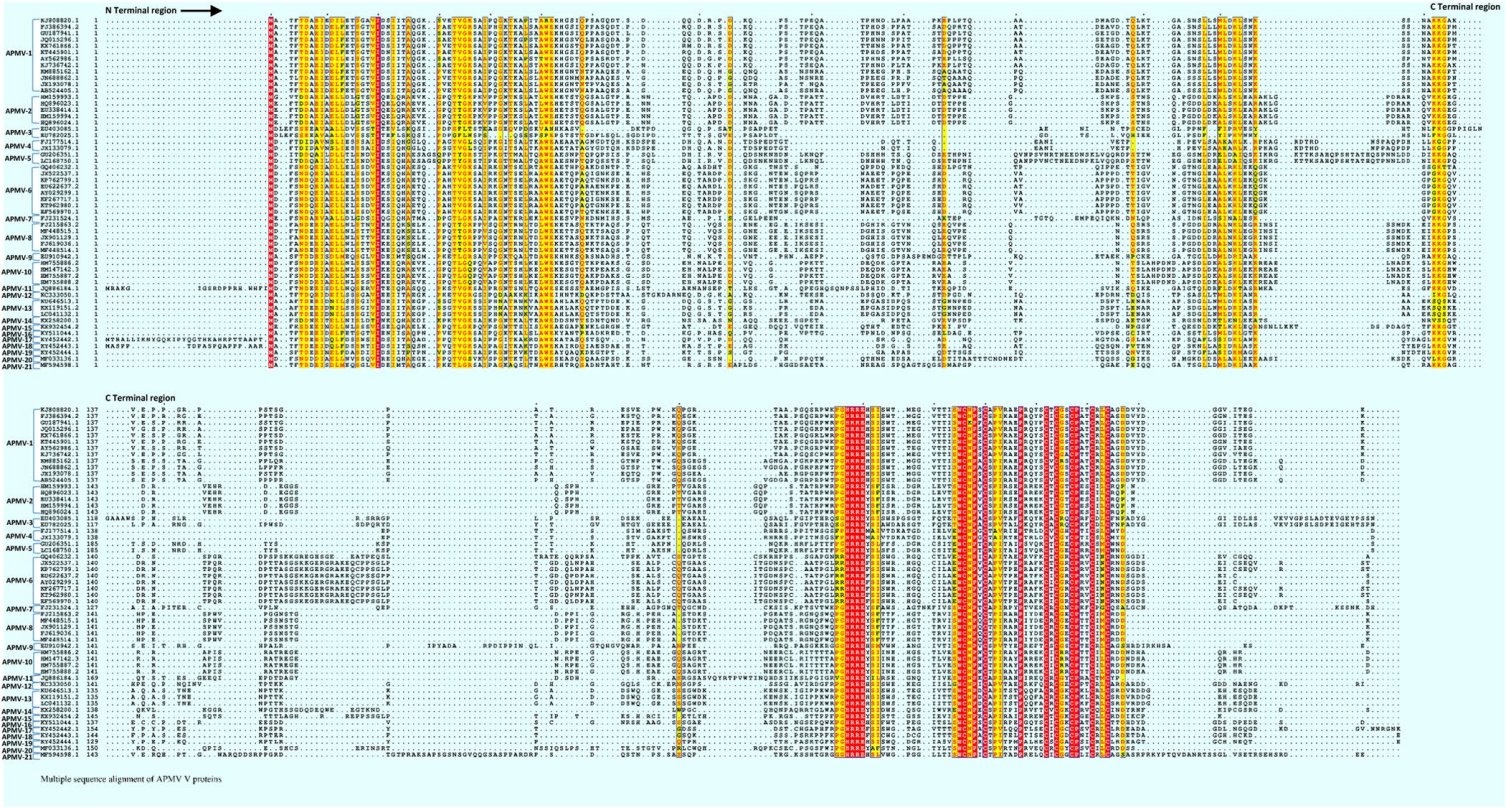
Multiple sequence alignment of V proteins of 21 APMV species. The conserved motifs and conserved amino acids are highlighted. The dots represent the gaps in the alignment. Highly conserved motifs and amino acids are in red and highlighted. The C terminal region is considered beyond the conserved KKG motif.

The multiple sequence alignment of V protein sequences of APMV species revealed higher amino acid conservations in both N (majorly in the first 60 amino acids) and C terminal regions (Fig. 1). All viruses in this study had the following conserved motifs similar to V proteins of other known paramyxoviruses (i) *KKG* motif in the N terminal region, which is the coding sequences at the *P* gene mRNA editing site (residues 132-134, corresponding to APMV-1 strain KJ808820.1, the first strain in the alignment file) except in APMV-3, APMV-4, APMV-12, APMV-13, APMV-14 and APMV-20 (ii) *HRRE* motif (residues 177 – 180, corresponding to APMV-1 strain KJ808820.1), (iii) *WCNP* motif (residues 195-198, corresponding to APMV-1 strain KJ808820.1) and (iv) conserved seven cysteine-rich domain. Interestingly, the following amino acids were also conserved in the C terminal region in majority of APMV species with few exceptions: Proline residues at five positions: (a) position 175 in ten species (except in APMV -3,-4,-6, -7, -8, -9, -10, -11, -15, -20 and -21), (b) position 198 in all APMVs, (c) position 202 (except in APMV-4), (d) position 207 in all APMVs and (e) position 218 in all APMVs; Glycine residues at three positions: (a) position 176 (except in APMV-4 and -6), (b) position 188 (except in certain strains of APMV-1 and APMV-7, -9, -11, -12, -13, -14, -15, -16, APV -A and -B), (c) position 215 (except in a single lentogenic strain, KM885162, of APMV-1, all strains of APMV-3, all strains of APMV-10 and interestingly in all these viruses, the glycine residue was replaced with arginine residue); Serine residues at two positions: (a) position 182 (except in APMV -4, -11, -20) and (b) position 194 (except in APMV -3, -4, -5, -6, -12, -14); Arginine residue at position 208 (except in APMV -3, -4, -5, -7, -8, -11, -13, -16, APV-A, -B and –C); Leucine residue at position 223 (except in APMV-5, -6, -7, -8 and -11) and Aspartic acid residue at position 227 (except in APMV-2, -3, -7, -9, -11 and -21). The percentage amino acid conservation in the C terminal region of V proteins of all 21 APMV species was between 30% (in the longest V protein, that of APMV-21) and 48% (in the shortest V protein, that of APV-C). The NLS were predicted in V proteins of APMV-5 and APMV-20 by cNLS mapper with a cut off score of 5.0. The NES were identified only in APMV-5 strains (Table 3b).

**Table 3:** Predicted nuclear localization (NLS) and nuclear export signals (NES) in W (3a) and V (3b) proteins of 21 APMV species.

APMV species	Genbank ID	Predicted Nuclear localization signal sequence and position in W proteins	Predicted Nuclear export signal sequence and positionin W proteins
**3a: NLS and NES within W proteins**			
APMV -1	FJ386394.2	^138^RAPKRGTTNVRLNSREVNPAAETVRKDRRTK^168^	^210^IPLYL^214^ (predicted by NetNES 1.1 Server); ^105^VDTQLRTGASNSLLL^119^,^202^SDQGRAKTIPLYLRI^216^, ^203^DQGRAKTIPLYLRIM^217^ (predicted by LocNES)
	JQ015296.1	^106^DTQLKTGASNSLLSMLDKLSNKSSNAKKGAH^136^	None predicted
	KX761866.1	^106^DTQLKTGASNSLLSMLDKLSNKSSNAKKGAH^136^	^210^ILLHLWIMSSYLSTL^224^ (predicted by LocNES)
	KT445901.1	^138^RAPKRGTTNVRLNSREVNPAAETVRKDRRTK^168^	^210^IPLYL^214^ (predicted by NetNES 1.1 Server); ^105^VDTQLRTGASNSLLL^119^, ^202^SDQGRAKTIPLYLRI^216^, ^203^DQGRAKTIPLYLRIM^217^ (predicted by LocNES)
	AY562986.1	^106^DAQLKTGASNSLLSMLDKLSNKSPNTKKGAH^136^	^13^LEI^15^ (predicted by NetNES 1.1 Server); ^1^MATFTDAEIDDILEI^n^ (predicted by LocNES)
APMV-6	GQ406232.1	^125^RKLEKQGKSSGKTTADSSPGRDPSPSKKGAR^n^	^13^LELSSDVI^20^(predicted by NetNES 1.1 Server)
APMV-9	EU910942.1	^157^RRRKTRHPADQPTHRDTAWSPERPPSCQSR^186^	^12^LMEQSGLVI^20^ (predicted by NetNES 1.1 Server ^125^KEGATGGLLDMLDRI^139^ (predicted by LocNES)
APMV-20	MF033136.1	^117^KSNMGKDLDSALAKLEKKAASIKSDK^142^ (within N terminal region)	None predicted
**3b: NLS and NES within V proteins**			
**APMV species**	**Genbank ID**	**Predicted Nuclear localization signal sequence and position in V proteins**	**Predicted Nuclear export signal sequence and positionin V proteins**
APMV-5	GU206351.1	^197^PTTKHTAKPNQDRLSNQKRHR^217^	^4^TDDQAILDLLTL-N
	LC168750.1	^n^PTTKHTAKSNQDRLSNQKRHR^217^	^4^TDDQAILDLLTL-N
APMV-20		^117^KSNMGKDLDSALAKLEKKAASIKSDK^142^ (within N terminal region)	None predicted

### Comparison of W protein sequences of APMV species

The length of W protein varied between 125 and 227 amino acids (aa) with calculated molecular weights between 13.30 kDa and 24.38 kDa. APMV-3 strain Netherland and APMV-7 had the shortest W protein (125 aa) and two strains of APMV-1, mesogenic strain KX761866.1 and velogenic strain KJ808820.1 (227 aa) had the longest W protein.

**Figure 2:**
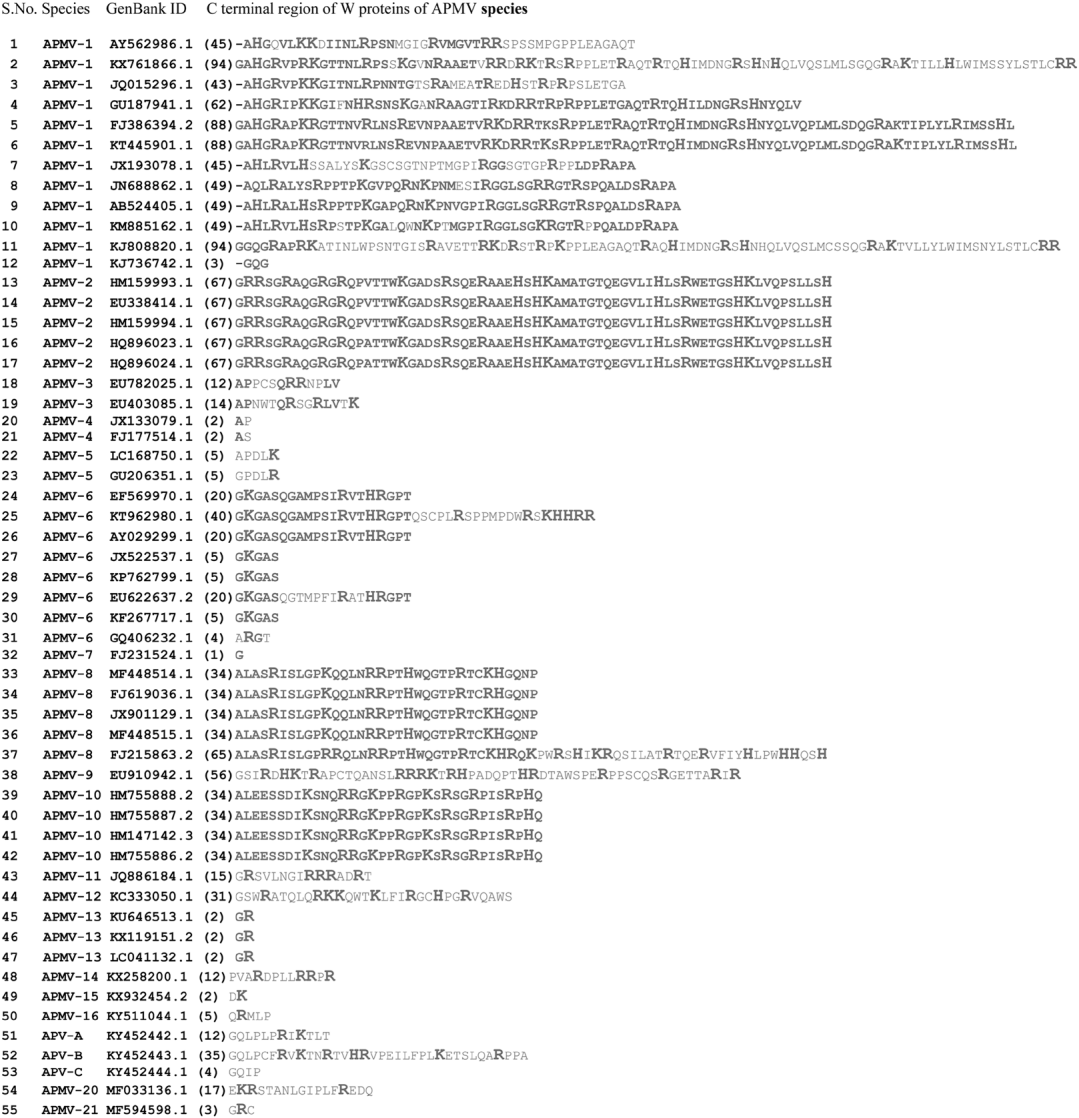
Comparison of C terminal region of W proteins of APMV species. The C terminal region is considered beyond the conserved KKG motif (please refer Fig. 1). Number of aminoacids in the unique C terminal is mentioned within parenthesis. The basic amino acids (R, K and H) are bolded and enlarged. Amino acid residues that are conserved between strains of the same species are bolded.

The length (in aa) of W protein was conserved among all the strains of APMV-2 (207 aa), all the strains of APMV-4 (137 aa), all the strains of APMV-5 (187 aa), all the strains of APMV-10 (172 aa) and all the strains of APMV-13 (150 aa). Also, similarity in the W protein length was noticed between the following species: W protein length of 172 aa was observed in all strains of APMV-10 and all strains of APMV-8 except strain FJ215863.2; W protein length of 177 aa was deduced in one strain of APMV-1 (JQ015296.1) and 3 strains of APMV-6 (EU622637.2, AY029299.1, EF569970.1), while the W protein length of APMV-12 and APMV-5 were 187 aa. Variations in the W protein length between strains within the same species were observed in APMV-1 (227, 221, 196, 183, 179, 177, 137 aa), APMV-3 (125, 127 aa), APMV-6 (157, 162, 177, 197 aa) and APMV-8 (172, 203 aa). APMV-1 strains analyzed in this study, had the longest unique C terminal region when compared to other APMV species (Table 1 and Fig. 2).

The lowest amino acid percentage identity (2.4) was observed between W proteins of APMV-12 and APMV-3 strain Netherland. Incidentally, APMV-3 strain Netherland also showed the lowest amino acid identity with W proteins of other APMV species. The lowest amino acid divergence (83.7) was noticed between APMV-1 isolate HN1007 (KX761866.1) and APMV-16, their W protein amino acid identity was 48.6% which was the highest homology observed between APMV species (Supplementary File S5).

The NLS were identified in five out of the twelve strains of APMV-1, in one of the eight strains of APMV-6 (GQ406232.1) and in APMV-9 in their C terminal region while NLS in W protein of APMV-20 was observed in the N terminal region (shared with P and V proteins). The presence of NES were predicted in these viruses except in one strain of APMV-1 (JQ015296.1) and APMV-20 (Table 3a).

### Phylogenetic tree and evolutionary distance analysis

**Figure 3:**
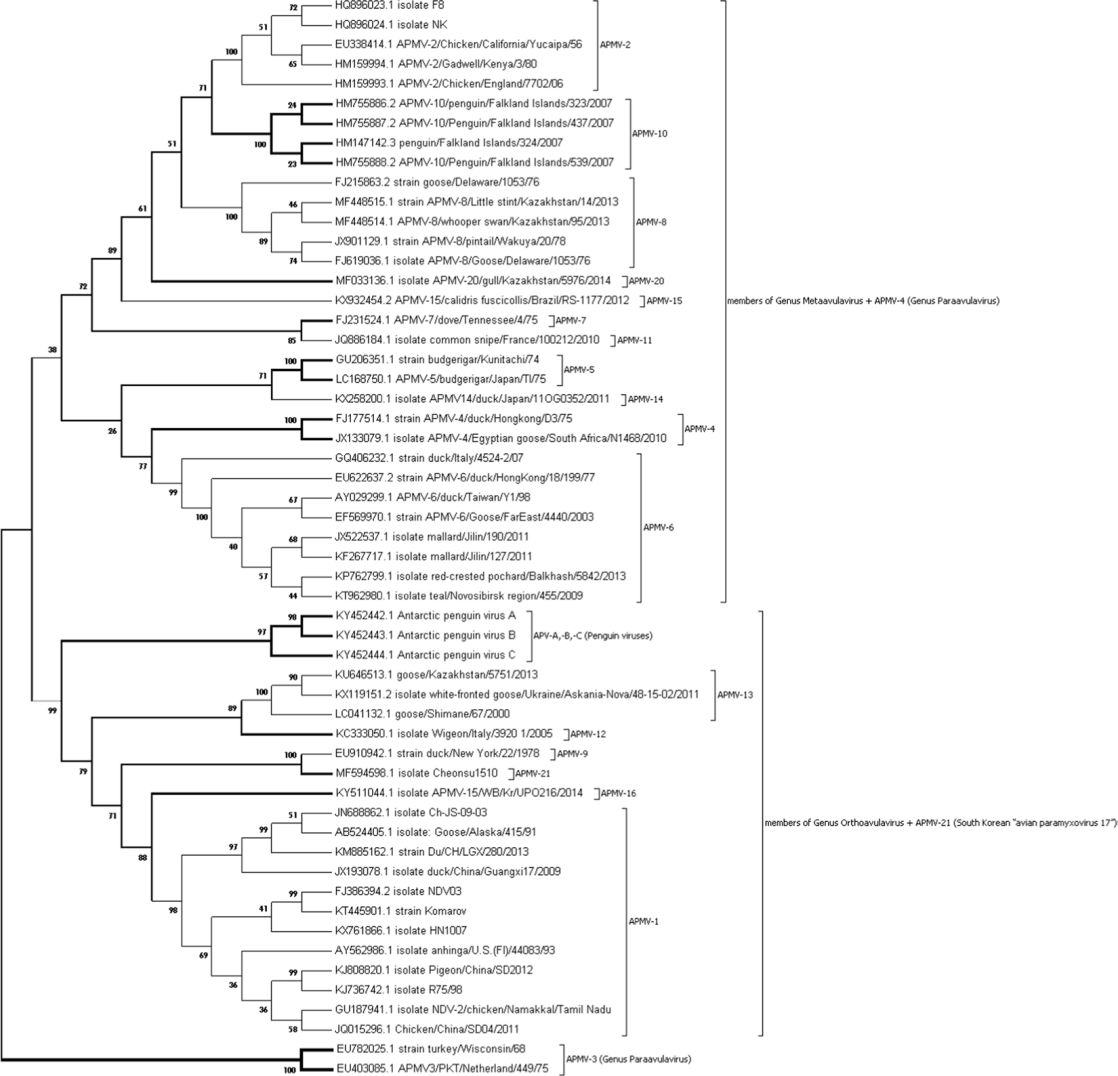
Phylogenetic tree derived from analysis of V proteins of APMV species by Maximum Likelihood method. The evolutionary history was inferred by using the Maximum Likelihood method based on the JTT matrix-based model. The bootstrap consensus tree inferred from 500 replicates is taken to represent the evolutionary history of the taxa analyzed. Evolutionary analyses were conducted in MEGA7. All the orthoavulaviruses clustered together, all metaavulaviruses grouped along with APMV-4 (Paraavulavirus) while APMV-3 strains (Paraavulavirus) formed a separate branch.

Based on their V protein sequences, phylogenetically the APMV species formed three distinct groups: group 1 consisted of APMV -3 strains, group 2 consisted of APMV -1, -9, -12, -13, -16, -21, APV -A, -B and -C (all orthoavulaviruses) and group 3 consisted of APMV -2, -5, -6, -7, -8, -10, -11, -14, -15, -20 and -4 (all metaavulaviruses and one paraavulavirus) (Fig. 3). The highest evolutionary divergence of 2.20 was observed between APMV-3 & APMV-4 and APMV-3 & APMV-12 followed by a divergence value of 1.91 between APMV-3 & APMV-5 and APMV-3 & APMV-11. The lowest divergence was between APV-A and APV-B (0.47) followed by APMV-9 & APMV-21 (0.50). The distance between the strains of the same species was noticed more in APMV-3 (0.4) followed by APMV-1 (0.297), which was further reiterated by their lower percentage of amino acid homology (Table 4).

**Table 4:**
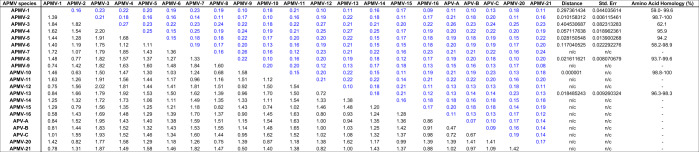
Estimates of Evolutionary Divergence over Sequence Pairs between Groups, analyzed for V proteins of 21 APMV species.

The number of amino acid substitutions per site from averaging over all sequence pairs between groups is shown. Standard error estimate(s) are shown above the diagonal. Analyses were conducted using the JTT matrix-based model. Evolutionary analyses were conducted in MEGA7.

**Figure 4:**
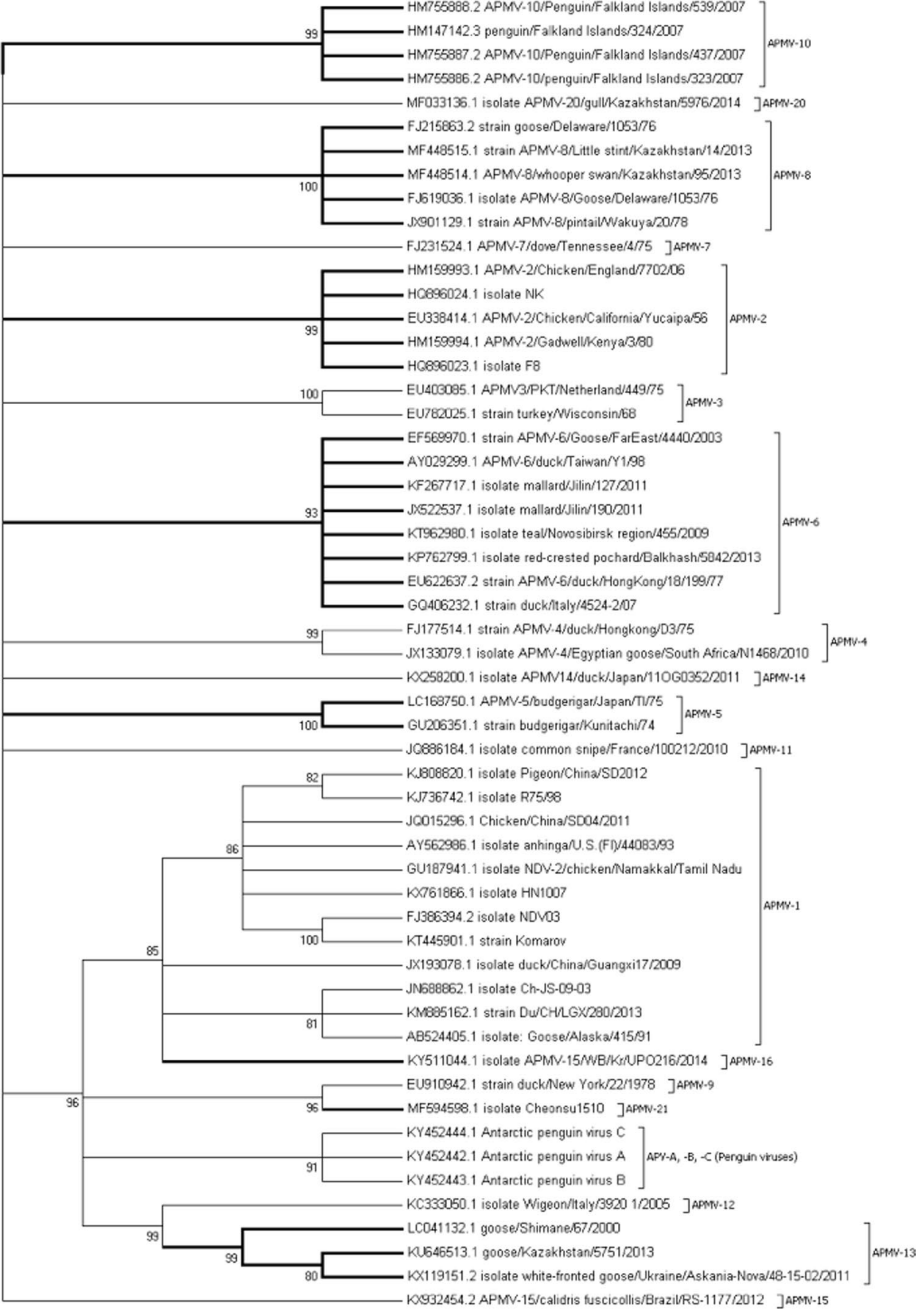
Phylogenetic tree derived from analysis of W proteins of APMV species by Maximum Likelihood method. The evolutionary history was inferred by using the Maximum Likelihood method based on the Dayhoff matrix based model. The bootstrap consensus tree inferred from 500 replicates is taken to represent the evolutionary history of the taxa analyzed. Evolutionary analyses were conducted in MEGA7.The tree shows clustering together of strains of the same species.

The phylogenetic tree obtained from W protein sequences analysis showed clustering of strains of the same species (Fig. 4). The evolutionary distance analyses of W proteins of APMV species revealed that APMV-3 species is more divergent than other APMV species. The highest evolutionary divergence was noticed between APMV-3 & APV-C species (10.322) followed by APMV-3 & APMV-13 (8.594) and APMV-3 & APMV-12 (8.270). The lowest divergence was observed between APMV-9 & APMV-21 (0.400) followed by APV-A & APV-B (0.407). The distance between the strains of the same species was more in APMV-3 (0.619) followed by APMV-1 (0.256) which was also apparent from their lower percentage of amino acid homology (Table 5).

**Table 5:**
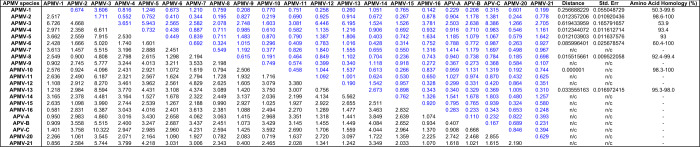
Estimates of Evolutionary Divergence over Sequence Pairs between Groups, analyzed for W proteins of 21 APMV species.

The number of amino acid substitutions per site from averaging over all sequence pairs between groups is shown. Standard error estimate(s) are shown above the diagonal. Analyses were conducted using the Dayhoff matrix based model. The rate variation among sites was modeled with a gamma distribution (shape parameter = 1). Evolutionary analyses were conducted in MEGA7.

### Selection pressure analysis

The dN/dS ratio was used to determine the natural selection pressure acting on the *P* gene edited products. The dN/dS ratio was estimated by DnaSP v6.12.03 for APMV species that comprised of more than one strain. The dN/dS values were significantly less than 1 for both V and W sequences (complete, N- and C-terminal regions) of most species explaining that they are under negative selection pressure. Only the C terminal region of V proteins of APMV-3 strains showed positive selection with dN/dS> 1 (Table 6).

**Table 6:** Selection Pressure Analysis of V and W proteins of 21 APMV species.

Species	V -complete dN/dS ratio	W -complete dN/dS ratio	P-V-W shared N terminal region dN/dS ratio	V -C terminal region dN/dS ratio	W -C terminal region dN/dS ratio
APMV-1	0.435	0.583	0.604	0.660	0.893
APMV-2	0.637	0.115	0.459	0.000	—
APMV-3	0.489	0.651	0.212	1.092	0.437
APMV-4	0.327	0.776	0.636	0.987	—
APMV-5	0.450	0.646	0.534	0.631	0.297
APMV-6	0.513	0.600	0.582	0.935	0.000
APMV-8	0.439	0.548	0.529	—	—
APMV-10	0.388	0.684	0.534	0.00	0.00
APMV-13	0.398	0.320	0.502	0.143	0.00

Species wise calculation of dN/dS ratios. Only those species comprising of more than one strain were included for the substitution calculation. The dN/dS ratio was estimated by DnaSP v6.12.03^74^. The dN/dS value was zero in those where the number of non-synonymous substitutions was zero and the dN/dS value could not be determined (−) in those where the number of synonymous substitutions were zero.

### Evolutionary rate analysis

The substitution rate of the V and W nucleotide sequences of APMV species that comprised of more than two strains were estimated by uncorrelated relaxed clock with lognormal using BEAST software. APMV-10 comprised of four strains, which were 98.65% to 100% identical to each other and hence the substitution rate could not be determined. The substitution rate was highest in APMV-13 followed by APMV-2, APMV-6 for both V and W proteins. The overall substitution rate was 7.37 × 10^−5^ for V protein and 8.07 × 10^−5^ for W protein (Table 7).

**Table 7:** Evolutionary Rate analysis by Molecular Clock- Estimated nucleotide substitution rates for V and W nucleotide sequences of all 21 APMV species.

Species	No. of strains	V protein substitution rate	W protein substitution rate
APMV-1	12	5.15 × 10^−4^	1.00 × 10^−4^
APMV-2	5	3.38 × 10^−2^	5.51 × 10^−2^
APMV-6	8	2.20 × 10^−2^	2.22 × 10^−2^
APMV-8	5	7.89 × 10^−3^	8.21 × 10^−3^
APMV-13	3	2.45 × 10^−2^	9.64 × 10^−2^
**Total**	**55 (21 APMV species)**	**7.37** × **10**^−**5**^	**8.07** × **10**^−**5**^

The best clock determined was uncorrelated relaxed clock with lognormal. The BEAST analysis could not done in species with less than 3 strains (APMV-3,-4,-5,-7,-9,-11,-12,-14,-15,-16,-17,-18,-19,-20 and -21) and data for APMV-10 could not be determined because APMV-10 strains were 98.65% to 100% identical to each other. The final data analysis was performed using tracer v 1.7.1 software.

## Discussion

Avian paramyxoviruses are known to infect a variety of bird species across the globe. Currently 21 species (previously called as serotypes) of APMVs are characterized and more viruses could be identified in future with improved viral surveillance programs. Paramyxoviruses with their small genome have a unique strategy of maximizing their genomic information by expressing viral proteins through co-transcriptional RNA editing. This helps to avoid error catastrophe caused by higher mutation rates often associated with larger genomes. Additionally, these viruses follow the ‘rule of six’ for efficient replication. Though, detailed studies on APMV structural genes and their complete genomes are available, a comparative knowledge of their accessory proteins expressed through RNA editing is lacking. In this study, using bioinformatics approach, we analyzed the *P* gene editing site, predicted and studied the protein sequences of edited products- V and W, of all 21 APMV species (55 viruses) known till date.

The hexamer phasing at the *P* gene editing site within each virus group is conserved^7^. We observed conserved hexamer phasing between certain APMV species and also, within each APMVs except in APMV-3 and for one strain of APMV-6. The hexamer phase is known to regulate the mRNA editing pattern, though subtle, it is important; for example, in human and bovine parainfluenza virus type 3 (PIV-3) in which the hexamer positions at *P* gene editing site are 2 to 5, higher mRNA editing frequency (~70%) and more number of G insertions (1 to 6 at equal frequencies) are observed while least mRNA editing ~30% with only 1 to 3 G insertions occur in Sendai virus wherein hexamer phase position is 1 at the *P* gene editing site ^7,79,80^. Based on the hexamer phasing position, it is anticipated that, in all APMVs except in APMV -20, the editing frequency could be extensive with possibilities of more number of G insertions. However, the cis-acting sequence of *P* gene editing site in APMV-20 (^3^’AA), suggests higher mRNA editing frequency and increased number of G insertions as reported in human and bovine PIV-3^81,82^. Thus APMVs seem to follow PIV-3 RNA editing phenotype.

Another interesting observation is the unique editing site sequence of APMV-11 (^3^’A_4_UUCU**U**C_5_), in which the unedited mRNA translates to V protein and it has been suggested that 2G insertions in mRNA translates to P protein^17^. In rubulaviruses with *P* gene editing site sequence of^3^’A_3_UUCUC_4_, realignment of the nascent mRNA/template hybrid during 1G insertion would mean non permissible A:C base pairing hence the minimum insertion expected is 2G^83^. The base pairing between the nascent chain and the template genome to form a hybrid is important to prevent transcriptional slippage by the polymerase^80^. Similarly, in APMV-11, 1G and 2G insertions would lead to unstable A:C base pairing, hence 3G insertion (V protein) could be the minimum number of insertions expected, also, while a 4G insertion would translate to W protein, a 5G insertion would lead to P protein synthesis. It needs to be explored if APMV-11 expresses more V protein (from both unedited mRNA and 3G insertions) than other paramyxoviruses. Furthermore, it will be interesting to study if deletions in addition to G insertions could happen in APMV-11 and other APMVs with longer C runs at the editing site as described previously with recombinant Sendai virus and PIV-3 minigenomes^83^.

Three factors, the editing site (sequence and the length of C runs), the type of sequences immediately upstream of the editing site (cis-acting sequence) and the hexamer phase positions are known to decide the editing phenotype (i.e. number of G insertions, deletions and frequency of mRNA editing) which further can influence the virus pathogenicity^81,82,84,85^. Among APMVs, variations are observed in (i) *P* gene editing site, (ii) hexamer phase at the editing site and also (iii) the sequences immediately upstream of the editing site, all of which will determine the expression levels and relative proportions of P, V and W proteins in the APMVs which in turn could explain their differences in replication, pathogenicity and virus-host interactions.

With respect to the length and amino acid composition of V and W proteins, there were huge variations between species than within species, which was also reiterated by their dN/dS estimates. The V proteins were more conserved than W proteins. Higher sequence identity for V proteins was observed between the strains of the same species (exception was APMV-3) more often than between species. Phylogenetically, V protein analysis of APMV species grouped viruses similar to the individual gene-based phylogeny^2^, all the members of genus *Orthoavulavirus* clustered into one group and all members of genus *Metaavulvirus* along with one of the avian paraavulaviruses (APMV-4) formed the second group while the avian paraavulavirus, APMV-3, formed an outgroup. This was further affirmed by evolutionary distance analysis. The phylogenetic analysis and the evolutionary distance data of both V and W proteins clearly showed that APMV-3 strains are the most divergent.

The N terminal region of V and W proteins which is shared with P protein, showed highest conservation among APMVs. In their C terminal region, the V proteins of paramyxoviruses carry conserved arginine and isoleucine residues upstream of highly conserved seven cysteine residues (zinc binding domain), known to play important roles in MDA5 interference, STAT1 degradation and blocking interferon signaling to evade host immunity^86–88^. The V proteins of all 21 APMV species analyzed in this study had the seven cysteine residues and remarkably, many other amino acids were also conserved in their C terminal region. Though similar observations have been made earlier in other paramyxovirus V proteins, their functional importance is unknown yet^86^.

The *V* and *W* genes were found to be under negative selection pressure with dN/dS <1 in all the species. This shows the conserved nature of the non-structural viral proteins within the species and probably indicates their functional importance, which is yet to be completely explored. Furthermore, the substitution rates of APMV species determined by molecular clock was varying across the species and was higher between species than within species. The substitution rates for W proteins was higher than V proteins except for APMV-1, where more strains were available for comparison. Though the substitution rate was slightly higher for V nucleotide sequence (5.15 × 10^−4^) of APMV-1 compared to W nucleotide sequence (1.0 × 10^−4^), it did not lead to changes in amino acid sequence as evident from dN/dS ratio estimates suggesting negative selection pressure. The higher conservation of V protein sequence implies its significant role in virus biology such as replication, pathogenesis and immune evasion.

In contrast to V proteins, the APMV W proteins were highly disordered, showed little sequence conservation when compared to V proteins and their divergence values were higher when compared to V proteins. The evolutionary data analysis of W proteins suggested higher sequence identity among strains of same species and higher variability between species. There were no conserved sequences or motifs in the C terminal region of W proteins except that most them carried large number of basic amino acids suggesting W protein to be highly basic as described previously^12^. The exceptions were one strain of APMV-1 (KJ736742.1), APMV-4, APMV-7 and APV-C, and all of them had shorter W protein length. This genetic diversity seen in the W proteins may determine the degree of pathogenesis, variable interferon antagonistic activity and the wide host range exhibited by the APMV species.

The likelihood of W mRNA occurrence could be less than that of V mRNA, because of two unstable base pairing created by the two mismatches (2G) during polymerase stuttering. This skepticism becomes more compelling and doubts rise to whether W protein is expressed at all in those APMV species whose predicted W protein sequences have only fewer amino acid residues in their C terminal region- single (in APMV-7) or two (in APMV-4, APMV-13 & APMV-15) or three (in APMV-1 isolate R75/98) or four (in APMV-6, JX522537.1 & APV-C) or five (in APMV-5 & APMV-16) amino acids. However, equal or higher frequencies of insertions of 1G and 2G during RNA editing have been reported in certain paramyxoviruses such as Nipah virus and Bovine Parainfluenza virus type 3^89,90^.

The presence of W mRNA of APMV-1 was first accounted in 1993 with a frequency of about 10%^12^, and the W protein expression from APMV-1 lentogenic strain Clone 30 and from APMV-1 lentogenic strain La Sota and velogenic strain SG10 was recently confirmed^16,91]^. We had earlier shown that W protein of APMV-1 mesogenic strain Komarov compartmentalized in the nucleus using plasmid system^30^, while the same has also been documented during virus infection in cells in the above two studies. Here, we report NLS and NES of W proteins predicted only in certain APMV species, also, we could identify NLS only in five out of twelve strains of APMV-1 implying that the not all the W proteins of APMV-1 strains localize in the nucleus. The W protein sequence analysis of nearly 1000 strains of APMV-1 in our lab show variations in the W protein length between strains (unpublished data),which was also reported recently in an analysis of 286 strains of NDV^91^, furthermore, W proteins of only about 50% of the strains analyzed by us are predicted to localize into the nucleus (data not shown) leading us to speculate that these differences in W proteins can attribute to the wide spectrum of pathogenicity and virulence observed in Newcastle disease.

Among paramyxoviruses, the W protein of Nipah and Hendra viruses, are the most well characterized. The nuclear localization of W protein of Nipah virus was found to modify p53 expression and activity^92^, sequester inactive STAT1 within nucleus^93^, prevent IRF3 phosphorylation, inhibit IFN signaling mediated both by the virus and TLR-3^29^, modulate host immunity, influence the disease course and viral pathogenesis specifically neurovirulence^27,28,94^. Intriguingly, neither the lack of W protein nor its cytoplasmic localization in APMV-1 strain clone 30 had any effect on viral replication in cell culture^16^. Though no conserved motifs could be identified between the W proteins of APMVs and Nipah virus, it will be interesting to study if similar roles are executed by W proteins of any or all the APMV species. To our knowledge, this is the first comprehensive and comparative evolutionary study of the *P* gene edited accessory viral proteins of APMVs. The information obtained by this study will enable designing future studies to understand the specific functions of conserved motifs/amino acids of V and W proteins and decipher their evolutionary significance on the virus and as well as on the host.

## Supplementary information

Supplementary information S1

Supplementary information S2

Supplementary information S3

Supplementary information S4

Supplementary information S5
