# Publisher Correction: Molecular evolution and genetic variations of V and W proteins derived by RNA editing in Avian Paramyxoviruses

**DOI:** 10.1038/s41598-020-70782-9

**Published:** 2020-10-02

**Authors:** Pachineella Lakshmana Rao, Ravi Kumar Gandham, Madhuri Subbiah

**Affiliations:** National Institute of Animal Biotechnology, Hyderabad, 500032 Telangana India

Correction to: *Scientific Reports* 10.1038/s41598-020-66252-x, published online 12 June 2020

The original version of this Article contained incorrect versions of Table 4 and Table 5. These have now been corrected in the HTML and PDF versions of this Article.

Additionally, the original Article contained 7 Supplementary Information files which were incorrectly uploaded and all incorrectly labelled as 'Supplementary Information'. The 5 correct Supplementary Information files are now included and labelled S1–S5.

